# The robustness and generalizability of findings on spontaneous false belief sensitivity: a replication attempt

**DOI:** 10.1098/rsos.172273

**Published:** 2018-05-02

**Authors:** Tobias Schuwerk, Beate Priewasser, Beate Sodian, Josef Perner

**Affiliations:** 1 Department of Psychology, Ludwig-Maximilians-University, Munich, Germany; 2 Department of Psychology, University of Salzburg, Salzburg, Austria; 3 Centre for Cognitive Neuroscience, University of Salzburg, Salzburg, Austria

**Keywords:** anticipatory looking, eye tracking, false belief, implicit Theory of Mind, replication

## Abstract

Influential studies showed that 25-month-olds and neurotypical adults take an agent's false belief into account in their anticipatory looking patterns (Southgate *et al.* 2007 *Psychol. Sci.* **18**, 587–592 (doi:10.1111/j.1467-9280.2007.01944.x); Senju *et al.* 2009 *Science* **325**, 883–885 (doi:10.1126/science.1176170)). These findings constitute central pillars of current accounts distinguishing between implicit and explicit Theory of Mind. In our first experiment, which initially included a replication as well as two manipulations, we failed to replicate the original finding in 2- to 3-year-olds (*N* = 48). Therefore, we ran a second experiment with the sole purpose of seeing whether the effect can be found in an independent, tightly controlled, sufficiently powered and preregistered replication study. This replication attempt failed again in a sample of 25-month-olds (*N* = 78), but was successful in a sample of adults (*N* = 115). In all samples, a surprisingly high number of participants did not correctly anticipate the agent's action during the familiarization phase. This led to massive exclusion rates when adhering to the criteria of the original studies and strongly limits the interpretability of findings from the test phase. We discuss both the reliability of our replication attempts as well as the replicability of non-verbal anticipatory looking paradigms of implicit false belief sensitivity, in general.

## 1. Introduction

Children at around 4 years of age become able to explain and predict another agent's behaviour by imputing mental states such as false beliefs [1,2]. This milestone in Theory of Mind (ToM) development, measured via the child's response to a test question, is preceded by spontaneous appreciation of an agent's false belief. As indicated by their gaze behaviour, children younger than 4 take an agent's false belief into account, before they can answer the test question correctly [3,4].

This dissociation is the empirical basis of current theoretical accounts that distinguish between an implicit and an explicit ToM [5–8]. According to these accounts, children acquire an explicit ToM around age 4, which allows them to deliberately consider an agent's mental state to infer reasons for his or her action. Yet, already infants have an implicit sensitivity to others' minds, which enables them to process others’ mental states in a spontaneous, unconscious, fast, but rigid manner. It is supposed that—after the acquisition of an explicit ToM—both processing modes co-exist throughout life and are employed to flexibly process others' and one's own mental states.

To date, the nature of the early competence to implicitly process false beliefs is heavily disputed [9–12]. In this study, we started off with the aim of identifying determinants of implicit false belief sensitivity in young children. Because of its theoretical relevance, we employed the anticipatory looking paradigm by Southgate *et al*. [13]. In this eye-tracking version of a change-of-location false belief task, the authors showed that at 25 months old, children already proactively predict an agent's false belief-based action. Using the same paradigm, Senju *et al*. [14] found that, while neurotypical adults show the same spontaneous false belief sensitivity, adults with autism spectrum condition fail to systematically predict the agent's false belief-based action.

We re-modelled the stimuli of Southgate *et al*. [13] and added additional conditions to pursue our research questions. In brief, in our first experiment, we failed to replicate the original finding and so had no good basis for investigating the effect of our manipulations. Although we adhered very closely to Southgate *et al*.'s procedure, the introduction of the new conditions caused some minor deviation. This led us to conduct a second experiment for the sole goal to see whether the results by Southgate *et al*. with children and Senju *et al*. [14] with neurotypical adults can be found in a tightly controlled replication. Because the findings of both original studies constitute an essential pillar of current theories on implicit and explicit ToM, we aimed to find the reported effect of spontaneous false belief sensitivity in a sufficiently large sample of 25-month-old children and adults.

Our first experiment was an overly ambitious attempt at investigating one particularly relevant factor that helps to explain why young children show some sensitivity to an agent's false belief in their anticipatory looking for the agent's behaviour before they can give firm answers about the agent's behaviour.

Initial studies of anticipatory looking in false belief contexts used a slight variation of the traditional unexpected transfer false belief task [1]. For instance, Clements & Perner [3] acted out a story for children about the mouse Sam. Sam's quarters had two exits, A and B. In front of each, there was a container. If Sam needed something from a container, he would come out through the respective exit. This allowed monitoring of children's eye gaze in expectation of where Sam would exit (anticipatory looking) in addition to the traditional measure of asking children where Sam would come out. On the test trials, Sam mistakenly thought that his piece of cheese was still in Box A, when children knew that it had been moved to B. For young 3-year-olds, the two measures strongly dissociated. When told that Sam would soon appear to look for his cheese, the majority of these children looked at exit A. But, when asked, they said that Sam would come out at B.

The original study [3] also tested children below the age of 3 years and found no evidence for false belief processing in anticipatory looking. However, Southgate *et al*. [13] reported such sensitivity for children as young as 2 years with several interesting changes to the original study. (1) Instead of a story character exiting from different locations, a person watching part of the scene over a screen used her left or right hand to reach for the object through different doors in the screen. (2) Children were not told a story but had to infer and anticipate actions on the basis of what they observed: (a) that the person was reaching in order to retrieve the object from one of the boxes in front of each door and (b) the imminent action was signaled by the two doors flashing briefly, accompanied by a chime. (3) In the test conditions, the person observed the object was put inside one of the boxes, was distracted by a phone call, and did not witness how the object was moved briefly to the other box and then removed from the scene. This made it similar to the ‘disappear condition’ used by Wimmer & Perner [1], which led to better performance in the traditional verbal task.

The objective of our first experiment was to investigate the importance of factor (3), because an inability to inhibit the attraction of the object's actual location has been widely seen as the reason for young children's problems with the standard task [9,15,16]. We approached this goal by using Southgate *et al*.'s [13] paradigm and contrasted their disappear condition with one where the object remained in the last box. We also wanted to see whether children's looking might be swayed by surface features of the task when they have no rational basis to expect the hand to appear through one or the other door. Thus, we introduced a knowledge control (KC) condition where the person knew that the object had disappeared from the scene.

The objective of our second experiment was to replicate Southgate *et al*. [13] and Senju *et al*. [14] in a sufficiently powered experiment, following the method of the original studies as closely as possible. We reasoned that if spontaneous false belief sensitivity, indicated by anticipatory looking, is a robust and generalizable phenomenon in children and adults, we should find this previously observed effect.

## 2. Experiment 1

With the aim of gaining further insight into the structure and solidity of children's implicit sensitivity to false beliefs, we presented 2- to 4-year-olds with Southgate *et al*.'s [13] *false belief* condition (condition FB2 in the original paper)^1^ and added a *false belief present* (FBP) and a KC condition. All three conditions were based on the original procedure in which an actor watched a puppet storing an object in one of two containers. After the actor was distractedly looking away, the puppet first transferred the object from one container to another, but then left the scene with it. In one condition, we exactly followed this original procedure and as the object was no longer in any container we called it *false belief disappear* (FBD) condition (the detailed description of the FBD condition can be found in electronic supplementary material, appendix A).

In the other two videos, we also followed the original procedure with the exception that the object did stay in the container after the transfer (FBP) or that the person witnessed the transfer and the removal and therefore knew that the object had disappeared (KC). In addition to gaze recording, we asked children an explicit question after they saw the false belief videos: ‘Where will the hand appear?’ The data of Experiment 1 is available on https://osf.io/feg6u/.

As the two new conditions could only play their intended role of clarifying the nature of early implicit ToM if the FBD condition had replicated, we refrain from further reporting them. However, due to the FBD task being part of a within subjects design some methodological details of those conditions need to be reported.

### 2.1. Method

#### 2.1.1. Participants

Forty-eight 2- to 3-year old children participated in the study (*M*_age_ = 31.7 months, range = 20.8–41.5, 25 female). Three additional children were tested but had to be excluded due to experimenter error (1), technical problems (1) or less than 20% gaze data recorded (1). Participants were recruited and tested in six different day-care centres in Salzburg, Austria. Parents gave informed written consent and the children received gifts for their participation.

#### 2.1.2. Exclusion criteria

We applied the same exclusion criteria as described by Southgate *et al*. [13]: (1) no correct anticipation of action by the second familiarization trial, (2) looked away at the crucial moment of the test trial and (3) did not look at either door on the test trial.

#### 2.1.3. Design and stimuli

**Figure 1.**
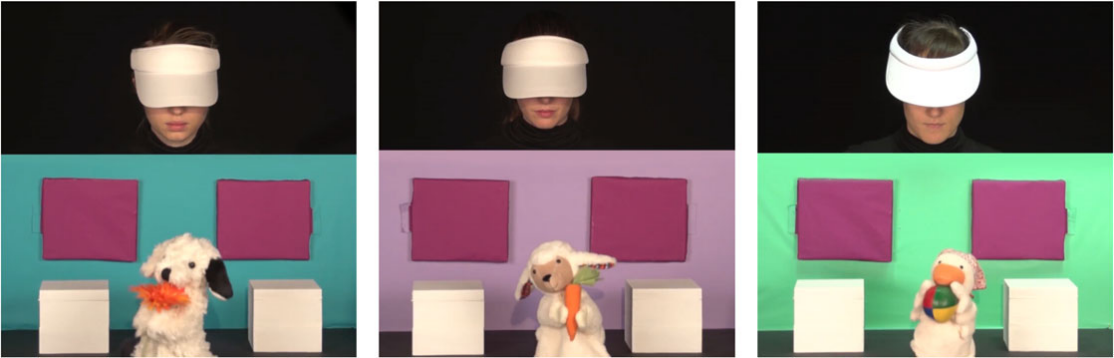
Experiment 1: stimuli examples. Children originally participated in three experimental conditions, each presented in a different setting (actress, agent, toy, colour of stage).

In a within-participants design, each child saw three videos—one per condition—in a partially counterbalanced order. While the position of the two false belief conditions was fully counterbalanced, the control condition was always presented as the second video. Each condition was presented in one out of three video settings that otherwise differed only in superficial elements. The colour of the stage (purple, green, blue), puppet (sheep, dog, goose), object (carrot, flower, ball) and actress (three different actresses, figure 1) were kept constant in each set. Condition (FBD, FBP and KC) and direction of transfer^2^ in the test trial (right to left/left to right) were counterbalanced.

Overall, 18 videos (six conditions in three different settings) were produced in the AV-Studio of the University of Salzburg. We modelled the stimuli closely to the original material by Southgate *et al*. [13]. Analogous to Southgate *et al*. [13], two familiarization trials preceded the test trial in each condition. The familiarization trials served to acquaint children to an audio-visual cue (chime and flashing of doors, approx. 1 s long), which preceded the agents' reaching action, and to make them understand that the agent is reaching for the object. The time window for gaze data analysis started at the onset of the cue and ended 1750 ms later. Our test trials differed from Southgate *et al*.'s [13] procedure in so far as they did not show the actor's reaching action to allow for an explicit question and to avoid influencing children's response on the subsequent trials. In the test trial, the video stopped 1750 ms after the offset of the cue and was replaced by an image of the last frame. In both false belief conditions, children were then asked by the experimenter to tell or indicate on the screen ‘where the hand will appear’. Choices of the door congruent with the actors' false belief were coded as correct answers, choices of the incongruent door as incorrect answers.

#### 2.1.4. Apparatus and procedure

We used a Tobii Pro X2-60 eye tracker (60 Hz sampling rate) [17]. The participants sat on a chair in front of the integrated 23-inch TFT screen (1600 × 900 pixel) at a distance of approximately 60 cm. Stimuli were presented and gaze was recorded with Tobii Studio 3.3.1 [17]. The eye tracker's position was individually adjusted via a flexible monitor arm. Children sat on their teacher's lap and completed the inbuilt 5-point calibration procedure before the experiment started.

#### 2.1.5. Data analysis and measures

Fixations were defined using the standard fixation filter of Tobii Studio 3.2 (velocity threshold: 35 pixels/door; distance threshold: 35 pixels). To extract gaze data of the anticipatory phase in the familiarization and test trials, we defined (1) time segments and (2) areas of interest (left- and right-hand door). We refer to the belief-congruent door as correct door and the other door as incorrect door. Tobii Studio statistics tool was used to extract two measures of interest: the location of first fixation and fixation duration on each door. We added a differential looking score (DLS) as a third measure. The DLS was calculated for fixation durations in the total anticipatory period. Therefore, the sum of the total duration of fixations on the incorrect door was subtracted from the sum of the total fixation duration of fixations on the correct door. This was then divided by the sum of the total duration of fixations on both doors. For statistical analyses, IBM SPSS Statistics 22 [18] was used. The significance level for all analyses was *p* ≤ 0.05, two tailed. For a detailed description of the analysis, see electronic supplementary material, appendix B.

### 2.2. Results

#### 2.2.1. Confirmatory analysis

We follow the original analysis by Southgate *et al*. [13]. Out of 48 children, 28 children (58.4%) had to be excluded because they did not anticipate the actor's action in the second familiarization trial (27, with 17 looking at the incorrect door) or did not look at either door on the test trial (1). To analyse whether an overall low interest in our task implementation could account for the low correct action anticipation rates in the second familiarization trial, we checked if participants who failed to meet this criterion had lower overall gaze rates (% gaze data recorded throughout the whole session) when compared with those who correctly anticipated the action in the second familiarization trial.

Mean percentage of overall gaze data recorded throughout the whole session did not differ between children who anticipated correctly (*M* = 78.2%, s.d. = 17.5%) and those who anticipated incorrectly (*M* = 84.6%, s.d. = 11.8%, *t*_46_ = 1.52, *p* = 0.134, Cohen's *d* = 0.43). The following analysis is based on the final sample of 20 children (*M*_age_ = 32.8 months, s.d. = 4.7, 13 female). Seven children directed their first fixation towards the correct door (*p* = 0.263, binomial test, chance level of 0.5). No difference in performance was found regarding the direction of transfer (first/last placement of ball in left/right box, *p* = 1, Fisher's exact test), task order (first or third position, *p* = 0.356, Fisher's exact test) or context (version of video, *χ*^2^ (2, *N* = 20) = 0.32, *p* = 0.851, *Φ*_Cramer_ = 0.13).

We further analysed looking time for each door during the anticipatory period. A repeated measures ANOVA with door (correct versus incorrect) as a within-participant factor showed no difference in looking times towards the correct (*M* = 343 ms, s.d. = 425 ms) when compared with the incorrect door (*M* = 542 ms, s.d. = 379 ms), *F*_1,19_ = 1.51, *p* = 0.233, $\eta_{p}^{2}=0.07$. No significant interaction effects were found when adding direction of transfer, *F*_1,18_ = 3.56, *p* = 0.076, $\eta_{p}^{2}=0.17,$ task order,^3^ *F*_1,18_ = 0.24, *p* = 0.631, $\eta_{p}^{2}=0.01,$ or context, *F*_2,17_ = 0.27, *p* = 0.768, $\eta_{p}^{2}=0.08,$ as a between-participants factor.

To test without carry-over effects from prior tasks, we repeated the analysis with those 10 children who had the FBD condition as the first task in the session. In this subsample 2, children directed their first fixation towards the correct door (*p* = 0.344, binomial test, chance level of 0.5). No difference in performance was found regarding the direction of transfer (*p* = 1, Fisher's exact test) or context, *χ*^2^ (2, *N* = 10) = 0.08, *p* = 0.961, *Φ*_Cramer_ = 0.09. The repeated measures ANOVA showed a non-significant result when comparing looking time to the incorrect (*M* = 464 ms, s.d. = 362 ms) and correct door (*M* = 346 ms, s.d. = 357 ms), *F*_1,9_ = 0.38, *p* = 0.552, $\eta_{p}^{2}=0.04$. Again, no significant effect was found when adding direction of transfer or context as a between-participants factor, *F*_1,8_ = 1.60, *p* = 0.241, $\eta_{p}^{2}=0.17;$ *F*_1,7_ = 0.124, *p* = 0.885, $\eta_{p}^{2}=0.03,$ respectively.

#### 2.2.2. Differential looking score

Although the one-sample *t*-test against zero showed a tendency for longer looking at the incorrect door, the mean DLS of −0.33 (s.d. = 0.74) did not differ significantly from chance, *t*_19_ = −1.98, *p* = 0.062, Cohen's *d* = 0.44. In the subsample of those children who watched the FBD condition first, the DLS (*M *= −0.29, s.d. = 0.75) did again not differ from chance, *t*_9_ = −1.23, *p* = 0.25, Cohen's *d* = 0.39.

#### 2.2.3. Explicit question

Out of the 20 children included in the final sample seven did not respond to the explicit question, seven indicated the incorrect and six the correct door. No association was found between explicit answers and the first fixation in the test trial (*p* = 0.592, Fisher's exact test). The interaction of the within-participants variable looking times and the between-participants variable explicit answer correct/incorrect was non-significant, *F*_1,11_ = 1.66, *p* = 0.224, $\eta_{p}^{2}=0.13$.

#### 2.2.4. Analysis of familiarization trials for all conditions

To measure understanding of the agent's false belief, it is imperative that the familiarization phase makes clear to children that the agent wants the object and will reach for it under standard conditions, i.e. no false belief. This condition is met if children anticipate the agent's action correctly on familiarization trials. To see whether any children had this basic understanding, we looked at familiarizations of all three conditions. Across the six familiarization trials, participants did not show above chance looking to the correct door (*p*-values ranging between 0.135 and 1, binomial test, chance level of 0.5), nor did they show significant improvement across the six trials, Cochran's *Q* (5)= 8.194, *p* = 0.146. Nevertheless, we analysed the false belief test data (FBD) of those eight children who anticipated correctly on more than three familiarization trials. Only three children anticipated correctly on test and two of them also looked longer at the correct window. Still, one could argue that no systematic anticipatory looking should be expected for the first familiarization trials of each condition because each condition used new materials and characters. Therefore, we looked at the performance in the false belief trials of those six children who showed correct anticipatory looking in all three of the second familiarization trials (complete gaze data for all second familiarizations as well as for the FBD test trial were available for overall 28 children). In the test trial of the FBD condition, only one child anticipated correctly whereas five children looked to the incorrect window. Results for fixation duration were the same. This suggests that children, who are most likely to have understood that the agent wants to reach for the object, focus on the window above the last location of the object in the test trial.

### 2.3. Discussion

As in our sample, compared to Southgate *et al*. [13], fewer children showed reliable anticipatory looking in the familiarization trials we need to consider potential factors accounting for this discrepancy. There are small deviations in our video material. First, we did not freeze the picture of the actor in the crucial moment of the test trial.^4^ Although 10 naive adults' ratings of the final scene about which of the two doors the person would open did not deviate from chance in any of the six FBD videos (*p*'s ranging between 0.754 and 0.109), we cannot entirely rule out the possibility that subtle motion cues of the actor could have influenced children's gaze.

Second, when checking the familiarization trials, we found that the time intervals between onset of the chime/flash and opening of the door varied between 1903 and 3313 ms. In Southgate *et al*. [13], this period was exactly timed to 2750 ms (1-second-long chime/flash plus 1750 ms until the opening of the door). It is therefore possible that the unreliable time intervals within familiarizations impeded the process of forming an association between the audio-visual cue and the action. Another possibility is that some children might have formed such an association for a longer delay and therefore they did not show anticipatory looks in the critical period in the test trial (within 1750 ms after the onset of the chime).

## 3. Experiment 2

Experiment 2 is a replication attempt adhering to a protocol for replication studies by Brandt *et al*. [19]. We tried to replicate previous findings on spontaneous false belief attribution in a sample of 25-month-old children [13] and in a sample of healthy adults [14]. We aimed at sample sizes that are 2.5 times larger than those of the original studies. The aspired number of participants included in the final analysis was at least *N* = 50 for the sample of 25-month-olds and *N* = 43 for the adult sample. We hereby followed the recommendation by Simonsohn [20] to protect previously observed effects from underpowered replication attempts. Experiment 2 was preregistered at the Open Science Framework (OSF). The preregistration protocol, stimuli, datasets and analysis protocols are available on https://osf.io/feg6u/. Following best practice recommendations, we reported how we determined our sample size, all data exclusions, all manipulations and all measures in the study [21].

### 3.1. Method

#### 3.1.1. Participants

A total of seventy-eight 25-month-old children took part in the study (*M*_age_ = 25 months 15 days, range = 23 months 23 days to 26 months 7 days; 39 female). They were recruited via birth records and received gifts for their participation. Their parents gave informed written consent and received monetary compensation for their participation. Seventy-one out of these children were tested at the Babylab at LMU Munich. The other seven children were tested at University of Salzburg. Note that this deviates from the preregistration protocol in which a final sample of 25 children should have been tested at each site. Owing to the unexpected high exclusion rates, we decided to stop data acquisition after an evaluation of the cost–benefit ratio (for details, see Results and Discussion).

The second sample comprised 115 adults (*M*_age_ = 23.1 years, range = 18–41; 47 female; due to an experimenter error, birthdates from two participants are missing). All participants were tested at LMU Munich. They were contacted via mailing lists or recruited in courses or directly on campus. They received monetary compensation.

#### 3.1.2. Exclusion criteria

We applied the same exclusion criteria to our samples as described by Southgate *et al*. [13]. Additionally, we had to exclude participants from the adult sample because they were familiar with the task or because they had a history of neurological or psychiatric condition (excluded before data analysis, table 1). Further, participants for whom less than 20% gaze data were recorded during the test session were excluded. If multiple reasons for exclusion were applicable to a participant, the criteria were assigned in the following order: (1) familiar with task, (2) history of neurological or psychiatric condition, (3) less than 20% gaze data were recorded, (4) no correct anticipation of action outcome by the second familiarization trial, (5) looked away at crucial moment of the test trial, (6) did not look at either door on the test trial.

**Table 1.** Experiment 2: number of included and excluded participants per sample, split for exclusion criteria.

	25-month-olds *N* (%)^a^	adults *N* (%)^a^
participants tested	78	115
included in final analyses	17 (21.8)	54 (47.0)
exclusion criteria^b^:		
(1) familiar with task	—	3 (2.6)
(2) history of neurological or psychiatric disorder	—	7 (6.1)
(3) less than 20% gaze data recorded	3 (3.8)	3 (2.6)
(4) no correct action anticipation by the 2nd fam. trial	51 (65.4)	29 (25.2)
(5) looked away at crucial moment of the test trial	1 (1.3)	—
(6) did not look at either window on the test trial	6 (7.7)	19 (16.5)

^a^Percentage of total number of tested participants.

^b^If multiple reasons for exclusion were applicable to a participant, the criteria were assigned in the order above.

#### 3.1.3. Stimuli

**Figure 2.**
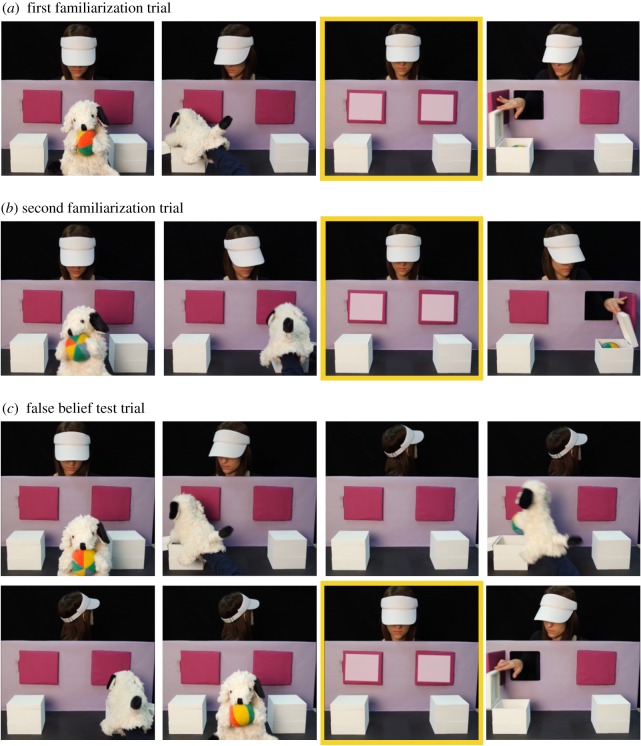
Experiment 2: trial overview. Still frames depicting key events in the first (*a*) and second (*b*) familiarization trial and in the false belief test trial (*c*). The still frames with the yellow border resemble the anticipatory period from which gaze data were obtained.

We modelled the stimuli as closely as possible to the original material by Southgate *et al*. [13]. Again, the original video examples served as templates for re-enacting the scene (figure 2). Based on a second-by-second transcript, we re-modelled the actions of the puppet and the actor. For details, see the transcript provided at https://osf.io/h5ptd/ and electronic supplementary material, appendix A. In stimulus preparation, we took care to present the same type and number of ostensive cues displayed by the puppet and the actor (e.g. direct gaze, waving and smiling).

This time, we tried to re-model the timings of the anticipatory phase as closely as possible. Just like in the original videos, we introduced a delay of 1750 ms from the offset of the audio-visual cue (chime and flashing of doors) to the opening of the door. Focusing on this period, we overlooked that our audio-visual cue was 2 s long instead of a duration of 1 s in the original stimuli. This, in combination with an inconsistency between timings of the original videos and their description in Southgate *et al*. [13],^5^ led to a deviation of our stimuli from the original ones. In Southgate *et al*.'s [13] videos, the children were familiarized with a 1 s long audio-visual cue, followed by a 1750 ms long delay phase until the observer opened one of the doors. Thus, they learned that 2.75 s after the onset of the audio-visual cue, the crucial action took place. In our videos, this interval was 3.75 s long (2 s long audio-visual cue plus 1750 ms delay phase). Yet, in both studies, the critical interval for the analysis of anticipatory looking was 1750 ms and began with the onset of the audio-visual cue. We evaluate a potential impact of this deviation on our results in the Discussion of Experiment 2.

To counterbalance the direction of transfer (left to right versus right to left), half of the participants watched a horizontally flipped version of the two familiarization trials and the test trial. Before each trial, an attention grabber was presented. In this 1 s long video the screen turned red and a chime (different from the one in the test trial) sounded.

Note that in the study by Senju *et al*. [14], participants saw two additional slightly varied familiarization trials to increase the likelihood that participants learn about the actor's goal to retrieve the ball and the contingency between the door flashing/chime and the subsequent opening of one of the doors. We reasoned that if two trials sufficiently familiarized 25-month-olds in the study by Southgate *et al*. [13], two familiarization trials would also be enough for our adult sample.

#### 3.1.4. Apparatus and procedure

The laboratory at LMU Munich used a Tobii T60 eye tracker (60 Hz sampling rate) [17]. The participants sat in front of the integrated 17-inch TFT screen (1280 × 1024 pixel) at a distance of approximately 60 cm. Stimuli were presented and gaze was recorded with Tobii Studio 3.2 [17]. The eye tracker's position was individually adjusted via a flexible monitor arm. The 25-month-old children sat on a high chair or on the lap of their parent (in that case the parent wore blackened sunglasses). Adult participants sat on a chair. Participants completed the inbuilt 5-point calibration procedure before the experiment started.

The laboratory at University of Salzburg used the same apparatus as described in Experiment 1. Procedures were identical between Salzburg and LMU with the exception that in Salzburg, instead of wearing glasses, the teacher or the parent was asked to close the eyes during calibration to ensure that the eye tracker recorded the child's and not the adult's gaze. Both laboratories used the same Tobii Studio project for stimulus presentation, data acquisition and data preprocessing.

#### 3.1.5. Data analysis and measures

The analysis of the eye-tracking data is identical to Experiment 1 and described in detail in electronic supplementary material, appendix B. In this experiment, we added differential looking score (DLS) as a third measure in addition to first fixation and looking time. The DLS was calculated for fixation durations in the total anticipatory period. Therefore, the sum of the total duration of fixations on the incorrect door was subtracted from the sum of the total fixation duration of fixations on the correct door. This was then divided by the sum of the total duration of fixations on both doors. For statistical analyses, IBM SPSS Statistics 24 [18] was used. The significance level for all analyses was *p* ≤ 0.05.

### 3.2. Results

#### 3.2.1. Confirmatory analysis

In this section, we report the analyses that correspond to those from the original papers by Southgate *et al*. [13] and Senju *et al*. [14]. A challenge for this replication attempt was the unexpected high exclusion rate of participants when criteria by Southgate *et al*. [13] were applied. From our adult sample, 53.0% had to be excluded from the final analysis. From the tested 25-month-old children, 78.2% had to be excluded. As we sought to have at least 50 25-month-olds and 43 adults in the final sample of included participants, we had to continue with testing participants until these sample sizes were met. These surprisingly high exclusion rates led to the decision to stop data acquisition for our sample of 25-month-olds. A total of 230 children would have been necessary to get to the final sample of 50 included participants. We reasoned that such a high exclusion rate precludes a solid interpretation of any results, irrespective of whether effects are replicated or not. Out of cost–benefit ratio evaluations, we refrained from testing additional 25-month-olds. Table 1 shows the number of included and excluded participants in each sample, split for each inclusion criterion. Remarkably, many participants had to be excluded because they did not anticipate the actor's action in the second familiarization trial (65.4% of tested 25-month-olds; 25.2% of tested adults). Analogously to Experiment 1, we checked if participants who failed to correctly anticipate the agent's action in the second familiarization trial had lower overall gaze rates than the finally included participants (calculated with the sample of the exploratory analysis, for details see below). For the 25-month-olds, this was not the case: children who passed (*M* = 87.6%, s.d. = 13.0%) and failed (*M* = 83.4%, s.d. = 17.1%) the original criterion did not differ in overall gaze rates, *t*_56_ = −0.90, *p* = 0.372, Cohen's *d* = −0.24. The same was found in the adult sample, *t*_73_ = 1.58, *p* = 0.118, Cohen's *d* = 0.37 (passed: *M* = 87.5%, s.d. = 10.9%; failed: *M* = 91.2%, s.d. = 8.3%).

##### 3.2.1.1. Twenty-five-month-olds

The confirmatory analysis is based on the final sample of 17 included children (*M*_age_ = 25 months 11 days, range = 23;23–26;5; eight female). In the anticipatory period, 6 out of these 17 children directed their first fixation towards the correct door (*p* = 0.332, binomial test, chance level of 0.5). We further analysed the looking time for each door during the anticipatory period. A repeated measures analysis of variance (ANOVA) with door (correct versus incorrect) as within-participants factor revealed that the 25-month-olds looked significantly longer to the incorrect door (*M* = 721 ms, s.d. = 541 ms) than to the correct door (*M* = 255 ms, s.d. = 721 ms), *F*_1,16_ = 6.67, *p* = 0.020, $\eta_{p}^{2}=0.30$. Finally, for the DLS, a one-sample *t*-test against zero showed that 25-month-old children had no looking bias towards the correct door. The mean DLS of −0.24 (s.d. = 0.83) did not differ significantly from chance, *t*_16_ = −1.20, *p* = 0.247, Cohen's *d* = 0.29. The electronic supplementary material provides additional analyses to check for side biases in gaze patterns, depending on the direction of transfer (which was counterbalanced between participants). In short, the three employed measures revealed no side bias in gaze behaviour of the final sample of 25-month-olds.

##### 3.2.1.2. Adults

**Figure 3.**
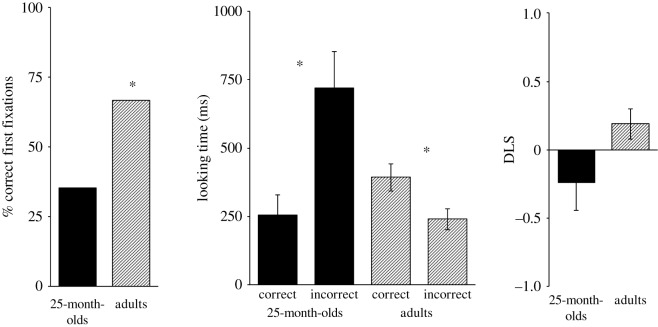
Experiment 2: results of confirmatory analysis. The graphs show means (±s.e.m.) for the three measures of interest, separated for 25-month-old children and adults. For location of first fixation, adults revealed a significant looking bias for the upcoming false belief-congruent action. They also looked significantly longer at the correct when compared with the incorrect door. By contrast, children looked significantly longer at the incorrect door. No significant effects were found for the DLS.

A total of 54 adults could be included in the final analysis (*M*_age_ = 23.3 years, range = 18–41; 21 female). Thirty-six out of these 54 participants correctly anticipated that the actor would open the door leading to the box in which she falsely believed that the ball would be located (*p* = 0.020, binomial test, chance level of 0.5). For looking times on the doors during the anticipatory period, a repeated measures ANOVA with the within-participants factor door (correct versus incorrect) showed a significant difference between looking times to the correct versus incorrect door, *F*_1,53_ = 4.36, *p* = 0.042, $\eta_{p}^{2}=0.76$. The included adults looked longer to the correct (*M* = 393 ms, s.d. = 365 ms) than to the incorrect door (*M* = 240 ms, s.d. = 290 ms). For the DLS (*M* = 0.19, s.d. = 0.83), a one-sample *t*-test against zero indicated a trend towards a significant looking bias towards the correct door, *t*_53_ = 1.71, *p* = 0.093, Cohen's *d* = 0.23. Figure 3 displays means for each sample and measure of the confirmatory analysis. Also our final adults sample showed no systematic preference for the left or right side of the screen (see the electronic supplementary material).

#### 3.2.2. Exploratory analysis

In this *post hoc* exploratory analysis, we included those 25-month-olds who did not correctly anticipate the actor's action in the second familiarization trial. We did this because Wang & Leslie [22] recently reported a replication of false belief-congruent anticipatory looking as reported by Southgate *et al*. [13] in a sample of 2- to 3-year-olds and adults where the original exclusion criterion was not applied.

##### 3.2.2.1. Twenty-five-month-olds

Including 25-month-olds who did not correctly anticipate the actor's action in the second familiarization trial, but fixated the door AOI(s) in the test trial, led to a sample size of 58 children for this analysis. Yet, also with this more lenient exclusion procedure, still only 28 out of these 58 children firstly fixated the correct door in the anticipatory period (*p* = 0.896, binomial test, chance level of 0.5). Their looking times on the doors did not significantly differ between the correct (*M* = 482 ms, s.d. = 423 ms) and incorrect door (*M* = 460 ms, s.d. = 457 ms), *F*_1,57_ = 0.05, *p* = 0.832, $\eta_{p}^{2}=0.001$ (repeated measures ANOVA with within-participants factor correct versus incorrect door). Furthermore, also the exploratory analysis of the DLS (*M* = 0.10, s.d. = 0.83) revealed no significant looking bias towards the correct door, *t*_57_ = 0.90, *p* = 0.374, Cohen's *d* = 0.12, one-sample *t*-test against zero.

##### 3.2.2.2. Adults

**Figure 4.**
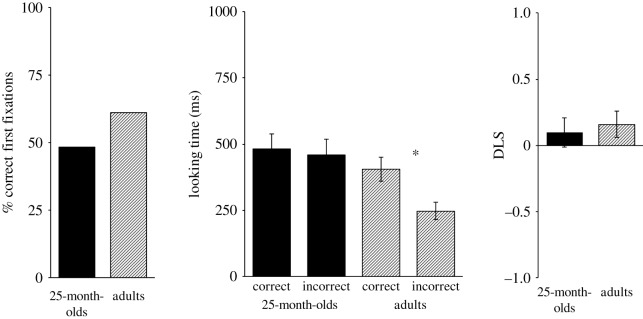
Experiment 2: results of exploratory analysis. In this analysis, also participants who did not correctly predict the agent's action in the second familiarization trial, but who fixated the door AOI(s) in the test trial, were included. In this analysis, adults looked significantly longer to the correct than to the incorrect door. In all other measures, no significant effects were observed.

Including adults who fixated the door(s) in the false belief test trial, but who did not predict the actor's action in the second familiarization trial, led to a sample of 75 participants. In this exploratory analysis, 46 out of the 75 adults directed their first fixation in the anticipatory period towards the correct door. Unlike in the confirmatory analysis, this distribution differed only marginally significantly from chance (*p* = 0.064, binomial test, chance level of 0.5). Consistent with the confirmatory analysis, the repeated measures ANOVA with door (correct versus incorrect) as within-participants factor yielded that adults looked significantly longer to the correct (*M* = 406 ms, s.d. = 391 ms) than to the incorrect door (*M* = 247 ms, s.d. = 285 ms), *F*_1,74_ = 6.07, *p* = 0.016, $\eta_{p}^{2}=0.08$. Finally, also in the exploratory analysis of the DLS, the one-sample *t-*test against zero indicated a marginally significant looking bias (*M* = 0.16, s.d. = 0.83) towards the correct door, *t*_74_ = 1.71, *p* = 0.092, Cohen's *d* = 0.20. Figure 4 displays means for each sample and measure of the exploratory analysis.

#### 3.2.3. Attention to observer

We additionally checked whether the participants' anticipatory looking was related to attention deployed to the observer whose belief they were supposed to track. It could be that, in contrast to other paradigms without a second agent (e.g. [23]), the interactive puppet pulled attention away from keeping track of the observer's visual access. It appears that this is possible because the puppet interacts in a conventional way with the audience, and participants may conceive of the procedure as an interactive puppet show, waiting for the puppet to continue the interaction, rather than keeping track of the observer's visual access and subsequent reaching actions.

If this were the case, we should find a relationship between the amount of fixations of the observer prior to the anticipatory period and our measures of interest in the test trial. We defined a time segment that started with the first frame the puppet appeared on the scene and ended with the last frame the puppet was visible before it left (57.8 s). For this segment, an AOI covering the observer was drawn (355 × 390 pixels). Subsequently, the number of fixations for this AOI and time interval was extracted as dependent variable. For 25-month-olds, a binary logistic regression with first fixation score as dichotomous outcome variable was performed. The number of fixations of the observer prior to the anticipatory period was entered as independent variable. The logistic regression model was not significant, *χ*^2^(1) = 0.03, *p* = 0.853 (Nagelkerke *R*^2^ < 0.01). The number of fixations to the observer (*M* = 13.9, s.d. = 8.0) could not predict whether 25-month-olds correctly anticipated her subsequent action. For the adults, an analogous binary logistic regression was performed. Also for this sample, the model was not significant, *χ*^2^(1) = 0.02, *p* = 0.964 (Nagelkerke *R*^2^ < 0.01). Thus also in adults, the number of fixations to the observer (*M* = 20.1, s.d. = 8.3) was not predictive for following false belief-congruent action anticipations.

### 3.3. Discussion

Experiment 2 was designed to answer the question whether we could replicate the effect of false belief sensitivity in 25-month-old children and adults adhering to the methods of the original studies as faithfully as possible. The short answer to this question is: yes, we were able to replicate the effect in adults; and no, we could not find the effect in 25-month-olds. However, the surprisingly high participant exclusion rates substantially limit the interpretability of these findings and therefore the long answer is more complicated.

A quarter of the adults and two-thirds of the 25-month-old children had to be excluded because they did not anticipate that the agent would open the door that led to the box with the ball by the second familiarization trial. Southgate *et al*. [13] reasoned that only participants who predicted the agent's action correctly in the second familiarization trial understood the agent's goal, the contingency between the flash/chime and the following opening of one of the doors, and were also motivated to engage in visual action anticipation. Following this criterion, Southgate *et al*. [13] had to exclude one-third of their tested 25-month-olds. In the study by Senju *et al*. [14], all adults correctly anticipated the agent's action by the last of four familiarization trials. Explanations for this striking difference between exclusion rates in the current and original studies are addressed in the General discussion.

The comparable overall gaze rates between those who passed and those who failed the original criterion speak against a potential objection that participants who failed the criterion merely were not interested enough in the task, indicated by reduced attention to the screen. In an exploratory analysis, we included participants who did not correctly predict the agent's action in the second familiarization trial, following a recent study by Wang & Leslie [22]. Yet, this procedure did not change the pattern of our results. In their study, Wang and Leslie reported that they replicated the effect of false belief-congruent action anticipation in a group of about 3-year-olds (mean age: 36.3 months) and adults. They used original stimulus material, but applied different exclusion criteria. Additional to including participants with incorrect action predictions in the second familiarization trial, they excluded children and adults who provided too little gaze data when compared with the respective group (three children from the bottom 5th percentile of total looking time to the doors; 17 adults from the bottom 25th percentile of total looking time to the doors). After this procedure, in the final samples, 19 out of 27 children and 28 out of 44 adults directed their first fixation towards the correct door in the test trial. These distributions are only significantly different from chance in a one-tailed binomial test. To be clear, we do not doubt the appropriateness of these criteria or of one-tailed testing of directional hypotheses. But, considering that there seemed a need for adopting new exclusion criteria seriously weakens Wang & Leslie's [22] evidence for false belief sensitivity in children and in adults.

We further checked whether participants in Experiment 2 attended more to the puppet on the stage than to the observer whose action they were supposed to anticipate and therefore failed the anticipatory looking measure for that reason. Yet, we found no predictive relationship between the amount of fixations directed towards the observer prior to the anticipatory period and subsequent fixations of the correct or incorrect door. In other words, participants who paid more visual attention to the observer were not more likely to perform better on the test trial. One motivation for this *post hoc* analysis was that there seemed to be an advantage of anticipatory looking procedures that use a self-propelled object, rather than an agent that hides an object [23–25]. However, evidence for false belief sensitivity observed in this paradigm does not seem to be overly robust either. For example, 7-year-old neurotypical children in Schuwerk *et al*. [25] showed above chance false belief-congruent action anticipations only in the DLS, but not when first fixations were considered.

A limitation of this replication attempt is a deviation of the original study in the timing of events during the crucial phase between the audio-visual cue that sought to elicit anticipatory looking and the to be anticipated action. In both studies, the anticipatory period started with the onset of the audio-visual cue (chime and flashing of doors) and lasted for 1750 ms. However, our audio-visual cue lasted 2 s and not 1 s as in the stimuli by Southgate *et al*. [13]. An alternative explanation why we failed to replicate the original findings could be that the time between the onset of the audio-visual cue and the observer's action was too long for 25-month-old children to build up an association between the cue and the action and thus our stimuli were not able to elicit anticipatory looking. Other studies that successfully found false belief-congruent anticipatory looking in infancy had delay phases (from the cue/disappearance of the agent to the to be anticipated action) between approximately 2 and 3 s [23,26,27]. However, there are also examples in the literature in which with such a delay phase no systematic false belief-congruent gaze behaviour was found [28]. Moreover, in a study by Surian & Geraci [29], the delay phase between the disappearance and reappearance of the agent was 3.5 s long and here already 17-month-olds successfully anticipated the false belief-congruent action. To conclude, we argue that it is unlikely that in our study the delay of 3.75 s was too long for 25-month-olds to learn the association between the cue and the action and therefore caused the high exclusion rates and the negative finding in the test trial. But, we cannot completely rule out the possibility that our comparably long interval contributed to the overall low correct anticipation rates in our experiment.

In sum, together with the current replication attempt, evidence for false belief sensitivity in anticipatory looking tasks is either absent [28] or present but weak [22,25,30]. In the General discussion, we address the reliability of our task implementation in comparison to previous studies and the role of exclusion criteria in anticipatory looking false belief tasks.

## 4. General discussion

In two experiments, we aimed to replicate Southgate *et al*.'s [13] finding that 25-month-old children seem to take an agent's false belief into account in their anticipatory looking patterns. With an adult sample, we further tried to replicate the finding by Senju *et al*. [14] that neurotypical adults systematically anticipate an agent's false belief-based action. In both experiments, we tested the false belief condition of the original studies (condition FB2 in the original papers), that is closer to classical object transfer false belief tests [3] and is less open to alternative explanations.

We failed to replicate the original findings in a sample of 2- to 3-year-olds (Experiment 1) and 25-month-olds (Experiment 2). In Experiment 1, no significant difference between looking to the false belief-congruent and the incorrect door was found. In Experiment 2, either no difference, or a significant looking bias towards the incorrect door was found, depending on the employed gaze measure. These findings fit well in the bigger picture of a number of other recent replication attempts of this paradigm [31–33] (for similar issues with other implicit false belief paradigms, see [34–36]). For example, Kulke *et al*. [33] could also not replicate the FB2 condition in a large sample of 2- to 6-year-old children. Further, Dörrenberg *et al*. [31] reported either a non-significant or a significant looking bias towards the incorrect door in 24-month-olds. Notably, all these studies employed the original material.

We replicated the original findings with our adult sample (Experiment 2). Our participants showed false belief-congruent anticipatory gaze behaviour in two out of three employed gaze measures. This adds to previous positive findings when examining adults with a non-verbal anticipatory looking false belief task [25,26,37] and might suggest that the phenomenon of false belief sensitivity indicated by anticipatory gaze behaviour might be more robust in older age groups. A recent longitudinal replication study by Grosse Wiesmann *et al*. [32] provides support for the idea that false belief-congruent action prediction is fragile in infancy, but becomes increasingly pronounced with age. However, our successful replication with adults has to be contrasted with other recent replication studies that did not find the effect in adults [38,39]. Taken together, the findings from these recent replication studies cast doubt on the robustness of implicit false belief sensitivity in children and adults.

Across all experiments, we encountered surprisingly high exclusion rates when the original criteria by Southgate *et al*. [13] were applied. Two-thirds of the children and a quarter of the adults failed to meet the inclusion criteria because they did not look systematically at the door through which the agent was going to grab the ball. That is, a large number of participants did not seem to build up the correct action anticipation that was suggested in this experimental procedure. This is worrying and questions the validity of the employed dependent variables. On logical grounds, we agree with the authors of the original studies that anticipating the agent's action at the end of the familiarization phase is a prerequisite for arguing that anticipatory looking on either door in the test trial is indicative of an action prediction that took the agent's belief into account.

Why did so many of our participants fail to correctly visually anticipate the agent's action in the second familiarization trial in Experiment 2? First, this could be due to certain aspects of this replication attempt, such as methodological differences in our task implementation or sample characteristics. Our procedure was close to the one described by Southgate *et al*. (refer to the preregistered replication receipt and stimuli description at https://osf.io/h5ptd/). Further, our sample of 2-year-olds had the exact same age as the original sample and it was large enough not to be underpowered [20]. Although the time interval for gaze data analysis was identical to the one in Southgate *et al*. [13], we inadvertently modelled the delay phase between the onset of the audio-visual and the false belief-based action 1 s longer. This deviation could serve as *post hoc* explanation for the high exclusion rates of Experiment 2, namely that the delay between the cue and the to be anticipated action was too long for our samples (especially the 25-month-olds) to learn this contingency. We argue that it is unlikely because this longer time interval is still comparable to previous research that reported evidence for false belief sensitivity in infancy [29]. Further, also with a shorter delay phase in Experiment 1, we observed these high exclusion rates and no false belief-congruent looking behaviour in the test trial. Moreover, other recent replication attempts also had high exclusion rates following the original criterion and could not replicate the original findings, although they employed the original material and the original delay-phase duration [31–33].

Second, the task itself, not specifically our implementation, could be an unreliable measure of false belief sensitivity. We argue that our findings, together with findings from previously published studies, suggest that the paradigm might not elicit belief-based anticipatory looking strongly enough. Rather, fixations by a considerable number of participants are randomly distributed or driven by some other cognitive process.

For example, the fact that Southgate *et al*. [13] had to exclude nearly one-third of the tested children due to a lack of action anticipation in the familiarization trial (11 out of 35), and that from the remaining 24 another 7 either looked at neither (*n* = 4, excluded from the final sample) or the wrong door (*n* = 3) in the test trial, points in this direction. Moreover, several other studies using this type of task provide no [28] or weak evidence for false belief sensitivity [22,25,30]. On the other hand, other versions of this paradigm seem to have been more successful in detecting false belief sensitivity, especially in adult samples [24,26,37].

Strikingly, the recently fast-growing body of replication attempts of these anticipatory looking paradigms could not help to elucidate this issue. For example, previous successful conceptual replications with children and adults [24,25] could in turn not be directly replicated [38]. In sum, the to date available replication attempts draw a complicated and mixed picture of replications, partial replications and non-replications [40].

There is, however, evidence on the predictive validity of an anticipatory looking measure from a longitudinal study which found significant correlations between taking false beliefs into account in an eye-tracking task at 18 months and explicit false belief understanding at 48 months [23] and the understanding of moral intentions at 60 months [41], independently of verbal IQ. Moreover, the anticipatory looking measure was significantly correlated with infants' performance in a goal-encoding habituation task [42] at seven months, suggesting coherence among measures of infant psychological reasoning [41]. These findings seem to indicate that a low-demand anticipatory looking measure may be valid to capture implicit false belief sensitivity, or some skill relevant for processing beliefs in infants.

Recently, another anticipatory looking paradigm version was published by Grosse Wiesmann *et al*. [30] (cf. [27]). In their paradigm, a cat follows a mouse through a tunnel that branches and has two exits. They measured if 3- to 4-year-old children correctly predicted which exit the cat would take based on its belief about the whereabouts of the mouse. An advantage of this paradigm is that it consists of multiple trials (10 familiarization trials, 12 false belief trials, six true belief trials) and might therefore produce more reliable results. Although evidence for false belief sensitivity is also rather weak (54% correct false belief trials in 3-year-olds and 4-year-olds), such a paradigm—if independently replicable—could be promising for future studies using visual false belief-based action predictions as a measure of individual competence. Apparently, the only anticipatory looking paradigm that has hitherto produced robust results is the one by Clements & Perner [3]. The false belief sensitivity assessed with this task in children between 3 and 5 years of age has been repeatedly documented in the same and independent laboratories [43–46]. Its robustness may be due to children being explicitly told what the story agent is after, whereas this has to be inferred in Southgate *et al*.'s [13] version.

Our findings further suggest that exclusion rates should be considered important for research questions, rather than just being an undesirable hassle during data acquisition and analysis. Different exclusion criteria used in available anticipatory looking studies [14,22,24,25,28,47] contributed to the confusion about whether infants, children and adults have an implicit sensitivity to false beliefs or not. We are convinced that, for each individual study, the exclusion of participants due to adjusted criteria may be necessary and justified. But, now that we have a bigger—and scattered—picture of the empirical basis of implicit false belief sensitivity in anticipatory looking paradigms, it is time to systematically investigate what the exclusion of children and adults means, instead of ignoring the reasons that drove their task performance and mentioning in a footnote that high exclusion rates are common in the study of infants. A systematic evaluation of this issue will advance our understanding of the reliability and validity of anticipatory looking paradigms to test false belief sensitivity. This, in turn, is a prerequisite for the development of strong theories on the nature of ToM.

In sum, the present findings, together with findings from previous studies, demonstrate that the reliability of replications of non-verbal anticipatory looking paradigms poses a serious challenge for ToM infant research. It seems that subtle differences in the implementation of anticipatory looking false belief tasks have a large impact on the obtained results. We do acknowledge the importance of painstakingly taking care that all critical methodological details are re-modelled in replication attempts. Further, we acknowledge the importance of context in replication attempts. As pointed out by Lucas [48], it can be argued that replications are even likely to fail because the psychological phenomenon of interest is dependent on a myriad of contextual factors not covered by the methods description of the original paper (e.g. laboratory setting, experimenter characteristics, time of the day, mood). But, theory-driven confirmatory research should be able to form and test predictions on psychological phenomena beyond such subtle methodological variations and contextual factors. Otherwise, theories—especially those that are driven by novel and exciting findings—become unfalsifiable.

We conclude that a systematic multi-laboratory replication project, agreeing on one appropriate anticipatory looking paradigm and standardized data processing, is required to assess the reliability of measuring implicit sensitivity to beliefs without the help of a verbal story in infants, children and adults.

## Supplementary Material

Supplementary Information

## Footnotes

1 In the original study by Southgate *et al*. [13]; same in Senju *et al*. [14] two versions of false belief test trials were tested. The false belief trial used in Experiments 1 and 2 corresponds to the false belief 2 condition of the original study. In the false belief 1 condition, the actor witnessed the transfer of the object from the left- to the right-hand box but did not witness its removal from the scene. In the false belief condition 2, the actor witnesses neither the transfer nor the removal. Because the false belief 2 condition is closer to the classical object transfer false belief test [1], because it is less open to alternative rule-based explanations of false belief-congruent looking behaviour, and because Southgate *et al*. [13] found no difference in anticipatory looking between those two versions, we decided to only implement false belief condition 2.

2 The objects’ first position (right versus left) alternated between first familiarization, second familiarization and test trial (left/right/left versus right/left/right).

3 The marginally significant interaction between direction of transfer and door results from children looking longer to the incorrect door when the object was transferred from the right- to the left-hand box (child's perspective) than from left to right. The electronic supplementary material includes a detailed analysis of the side bias.

4 This is not mentioned in Southgate *et al*.'s [13] paper. After watching the original videos frame by frame we detected this detail.

5 Unlike it seemed from the timing of the original videos, the 1750 ms long gaze data analysis interval started with the onset (and not the offset) of the audio-visual cue.

## Data accessibility

The data generated in this study are available at https://osf.io/feg6u/.

## References

[1] Wimmer H, Perner J 1983 Beliefs about beliefs: representation and constraining function of wrong beliefs in young children's understanding of deception. Cognition 13, 103–128. (doi:10.1016/0010-0277(83)90004-5) 6681741 10.1016/0010-0277(83)90004-5

[2] Wellman HM, Cross D, Watson J 2001 Meta-analysis of theory-of-mind development: the truth about false belief. Child Dev. 72, 655–684. (doi:10.1111/1467-8624.00304) 11405571 10.1111/1467-8624.00304

[3] Clements WA, Perner J 1994 Implicit understanding of belief. Cogn. Dev. 9, 377–395. (doi:10.1016/0885-2014(94)90012-4)

[4] Onishi KH, Baillargeon R 2005 Do 15-month-old infants understand false beliefs? Science 308, 255–258. (doi:10.1126/science.1107621) 15821091 10.1126/science.1107621 PMC3357322

[5] Apperly IA, Butterfill SA 2009 Do humans have two systems to track beliefs and belief-like states? Psychol. Rev. 116, 953–970. (doi:10.1037/a0016923) 19839692 10.1037/a0016923

[6] DeBruin LC, Newen A 2012 An association account of false belief understanding. Cognition 123, 240–259. (doi:10.1016/j.cognition.2011.12.016) 22297384 10.1016/j.cognition.2011.12.016

[7] Low J, Apperly IA, Butterfill SA, Rakoczy H 2016 Cognitive architecture of belief reasoning in children and adults: a primer on the two-systems account. Child Dev. Perspect. 10, 184–189. (doi:10.1111/cdep.12183)

[8] Perner J, Roessler J 2012 From infants' to children's appreciation of belief. Trends Cogn. Sci. 16, 519–525. (doi:10.1016/j.tics.2012.08.004) 22964134 10.1016/j.tics.2012.08.004 PMC3460239

[9] Baillargeon R, Scott RM, He Z 2010 False-belief understanding in infants. Trends Cogn. Sci. 14, 110–118. (doi:10.1016/j.tics.2009.12.006) 20106714 10.1016/j.tics.2009.12.006 PMC2930901

[10] Heyes C 2014 False belief in infancy: a fresh look. Dev. Sci. 17, 647–659. (doi:10.1111/desc.12148) 24666559 10.1111/desc.12148

[11] Perner J, Ruffman T 2005 Infants’ insight into the mind: how deep? Science 308, 214–216. (doi:10.1126/science.1111656) 15821079 10.1126/science.1111656

[12] Rakoczy H 2012 Do infants have a theory of mind? Br. J. Dev. Psychol. 30, 59–74. (doi:10.1111/j.2044-835X.2011.02061.x) 22429033 10.1111/j.2044-835X.2011.02061.x

[13] Southgate V, Senju A, Csibra G 2007 Action anticipation through attribution of false belief by 2-year-olds. Psychol. Sci. 18, 587–592. (doi:10.1111/j.1467-9280.2007.01944.x) 17614866 10.1111/j.1467-9280.2007.01944.x

[14] Senju A, Southgate V, White S, Frith U 2009 Mindblind eyes: an absence of spontaneous theory of mind in Asperger syndrome. Science 325, 883–885. (doi:10.1126/science.1176170) 19608858 10.1126/science.1176170

[15] Russell J, Mauthner N, Sharpe S, Tidswell T 1981 The ‘windows task’ as a measure of strategic deception in preschoolers and autistic subjects. Br. J. Dev. Psychol. 9, 331–349. (doi:10.1111/j.2044-835X.1991.tb00881.x)

[16] Setoh P, Scott RM, Baillargeon R 2016 Two-and-a-half-year-olds succeed at a traditional false-belief task with reduced processing demands. Proc. Natl Acad. Sci. USA 113, 13 360–13 365. (doi:10.1073/pnas.1609203113) 10.1073/pnas.1609203113 PMC5127346 27821728

[17] Tobii Technology. 2012 Tobii Studio computer software. Stockholm, Sweden: Tobii Technology.

[18] SPSS Inc. 2013 IBM SPSS Statistics computer software. Chicago, IL: SPSS Inc.

[19] Brandt MJ et al. 2014 The replication recipe: what makes for a convincing replication? J. Exp. Soc. Psychol. 50, 217–224. (doi:10.2139/ssrn.2283856)

[20] Simonsohn U 2015 Small telescopes detectability and the evaluation of replication results. Psychol. Sci. 26, 559–569. (doi:10.1177/0956797614567341) 25800521 10.1177/0956797614567341

[21] Simmons JP, Nelson LD, Simonsoh U 2012 A 21 word solution. Dialogue: Offic. Newslett. Soc. Person. Soc. Psychol. 26, 4–7. (doi:10.2139/ssrn.2160588)

[22] Wang L, Leslie AM 2016 Is implicit theory of mind the ‘real deal’? The own-belief/true-belief default in adults and young preschoolers. Mind Lang. 31, 147–176. (doi:10.1111/mila.12099)

[23] Thoermer C, Sodian B, Vuori M, Perst H, Kristen S 2012 Continuity from an implicit to an explicit understanding of false belief from infancy to preschool age. Br. J. Dev. Psychol. 30, 172–187. (doi:10.1111/j.2044-835X.2011.02067.x) 22429040 10.1111/j.2044-835X.2011.02067.x

[24] Schuwerk T, Vuori M, Sodian B 2015 Implicit and explicit theory of mind reasoning in autism spectrum disorders: the impact of experience. Autism 19, 459–468. (doi:10.1177/1362361314526004) 24627427 10.1177/1362361314526004

[25] Schuwerk T, Jarvers I, Vuori M, Sodian B 2016 Implicit mentalizing persists beyond early childhood and is profoundly impaired in children with autism spectrum condition. Front. Psychol. 7, 1696 (doi:10.3389/fpsyg.2016.01696) 27840620 10.3389/fpsyg.2016.01696 PMC5083838

[26] Low J, Watts J 2013 Attributing false beliefs about object identity reveals a signature blind spot in humans' efficient mind-reading system. Psychol. Sci. 24, 305–311. (doi:10.1177/0956797612451469) 23307943 10.1177/0956797612451469

[27] Meristo M, Morgan G, Geraci A, Iozzi L, Hjelmquist E, Surian L, Siegal M 2012 Belief attribution in deaf and hearing infants. Dev. Sci. 15, 633–640. (doi:10.1111/j.1467-7687.2012.01155.x) 22925511 10.1111/j.1467-7687.2012.01155.x

[28] Zmyj M, Prinz W, Daum MM 2015 Eighteen-month-olds’ memory interference and distraction in a modified A-not-B task is not associated with their anticipatory looking in a false-belief task. Front.Psychol. 6, 857 (doi:10.3389/fpsyg.2015.00857) 26157409 10.3389/fpsyg.2015.00857 PMC4475791

[29] Surian L, Geraci A 2012 Where will the triangle look for it? Attributing false beliefs to a geometric shape at 17 months. Br. J. Dev. Psychol. 30, 30–44. (10.1111/j.2044-835X.2011.02046.x) 22429031 10.1111/j.2044-835X.2011.02046.x

[30] Grosse WC, Friederici AD, Singer T, Steinbeis N 2017 Implicit and explicit false belief development in preschool children. Dev. Sci. 20, 1–15. (doi:10.1111/desc.12445) 10.1111/desc.12445 27696613

[31] Dörrenberg S, Rakoczy H, Liszkowski U In press. How (not) to measure infant theory of mind: testing the replicability and validity of four non-verbal measures. Cogn. Dev. (doi:10.1016/j.cogdev.2018.01.001)

[32] Wiesmann CG, Friederici AD, Disla D, Steinbeis N, Singer T In press. Longitudinal evidence for 4-year-olds' but not 2-and 3-year-olds’ false belief-related action anticipation. Cogn. Dev. (doi:10.1016/j.cogdev.2017.08.007) 10.1016/j.cogdev.2017.08.007 PMC6103291 30147231

[33] Kulke L, Reiß M, Krist H, Rakoczy H In press. How robust are anticipatory looking measures of Theory of Mind? Replication attempts across the life span. Cogn. Dev. (doi:10.1016/j.cogdev.2017.09.001)

[34] Powell LJ, Hobbs K, Bardis A, Carey S, Saxe R In press. Replications of implicit theory of mind tasks with varying representational demands. Cogn. Dev. (doi:10.1016/j.cogdev.2017.10.004)

[35] Priewasser B, Rafetseder E, Gargitter C, Perner J In press. Helping as an early indicator of a theory of mind: mentalism or teleology? Cogn. Dev. (doi:10.1016/j.cogdev.2017.08.002) 10.1016/j.cogdev.2017.08.002 PMC7099932 32226221

[36] Crivello C, Poulin-Dubois D In press. Infants' false belief understanding: a non-replication of the helping task. Cogn. Dev. (doi:10.1016/j.cogdev.2017.10.003)

[37] Schneider D, Bayliss AP, Becker SI, Dux PE 2012 Eye movements reveal sustained implicit processing of others’ mental states. J. Exp. Psychol. Gen. 141, 433–438. (doi:10.1037/a0025458) 21910557 10.1037/a0025458

[38] Burnside K, Ruel A, Azar N, Poulin-Dubois D In press. Implicit false belief across the lifespan: non-replication of an anticipatory looking task. Cogn. Dev. (doi:10.1016/j.cogdev.2017.08.006)

[39] Kulke L, von Duhn B, Schneider D, Rakoczy H In press. Is implicit theory of mind a real and robust phenomenon? Results from a systematic replication study. Psychol. Sci. 10.1177/0956797617747090 29659340

[40] Kulke L, Rakoczy H In press. Implicit theory of mind—an overview of current replications and non-replications. Data Brief. (doi:10.1016/j.dib.2017.11.016) 10.1016/j.dib.2017.11.016 PMC5694957 29188228

[41] Sodian B, Licata M, Kristen-Antonow S, Paulus M, Killen M, Woodward A 2016 Understanding of goals, beliefs, and desires predicts morally relevant theory of mind: a longitudinal investigation. Child Dev. 87, 1221–1232. (doi:10.1111/cdev.12533) 27091804 10.1111/cdev.12533

[42] Woodward AL 1998 Infants selectively encode the goal object of an actor's reach. Cognition 69, 1–34. (doi:10.1016/S0010-0277(98)00058-4) 9871370 10.1016/s0010-0277(98)00058-4

[43] Ruffman T, Garnham W, Import A, Connolly D 2001 Does eye gaze indicate implicit knowledge of false belief? Charting transitions in knowledge. J. Exp. Child Psychol. 80, 201–224. (doi:10.1006/jecp.2001.2633) 11583523 10.1006/jecp.2001.2633

[44] Garnham WA, Ruffman T 2001 Doesn't see, doesn't know: is anticipatory looking really related to understanding or belief? Dev. Sci. 4, 94–100. (doi:10.1111/1467-7687.00153)

[45] Low J 2010 Preschoolers' implicit and explicit false-belief understanding: relations with complex syntactical mastery. Child Dev. 81, 597–615. (doi:10.1111/j.1467-8624.2009.01418.x) 20438463 10.1111/j.1467-8624.2009.01418.x

[46] Wang B, Low J, Jing Z, Qinghua Q 2012 Chinese preschoolers’ implicit and explicit false-belief understanding. Br. J. Dev. Psychol. 30, 123–140. (doi:10.1111/j.2044-835X.2011.02052.x) 22429037 10.1111/j.2044-835X.2011.02052.x

[47] Senju A et al. 2010 Absence of spontaneous action anticipation by false belief attribution in children with autism spectrum disorder. Dev. Psychopathol. 22, 353–360. (doi:10.1017/S0954579410000106) 20423546 10.1017/S0954579410000106

[48] Lucas RE. (2017). http://deskreject.com/2017/06/w.w.p.m.d.

