# Supplementary material for: The robustness and generalizability of findings on spontaneous false belief sensitivity: a replication attempt
Source: R Soc Open Sci. 2018 May 2;5(5):172273. doi: 10.1098/rsos.172273 (PMC5990829; doi:10.1098/rsos.172273)
Supplement: Supplementary Information [file rsos172273supp1.docx]

**Supplementary Material**

**Appendix A: Detailed description of stimulus material of Experiment 1 and Experiment 2**

Videos started with a familiarization trial in which an actor watched a puppet appearing on the scene, carrying an object, e.g., a colorful ball. The puppet put the ball on the ground between two boxes, opened the left-hand box, placed the ball inside, closed the box, and left the scene. Subsequently, two doors through which the actor could reach for the boxes, flashed up. Simultaneously, a chime sounded. After that, the actor opened the left-hand door, reached for the ball in the left-hand box, took the ball and smiled at it. In the second familiarization trial the same events took place, except that the puppet placed the ball in the right-hand box.

In the test trial, the puppet appeared with the ball and placed it on the ground between the two boxes. It opened the left-hand box, put the ball inside, closed the box and left the scene. Subsequently a phone started ringing, attracting the actor’s attention away from the scene so that she did not witness the following events: the puppet re-entered the scene, opened the left-hand box, took the ball, placed it on the ground in the center of the scene, and closed the left-hand box. It then opened the right-hand box, placed the ball inside, closed the box and returned to the center of the scene. After that, it re-opened the right-hand box, took out the ball and put in on the ground between the two boxes. The puppet closed the right-hand box, took the ball and left the scene. Subsequently, the phone stopped ringing and the actor turned back to the scene. The doors flashed up, accompanied by a chime. The anticipatory phase for gaze pattern analysis started with the onset of the flash/chime and ended 1750 ms later. In Experiment 1 the video stopped and a still frame was presented while children were asked the explicit question. In Experiment 2 the actor opened the left-hand door to reach for the ball in the left-hand box in which she falsely believed it would be located. Half of the participants watched horizontally flipped versions of the two familiarization trials and the test trial were presented to counterbalance for direction of transfer.

**Appendix B: Data analysis and measures**

Fixations were defined using the standard fixation filter of Tobii Studio 3.2 (velocity threshold: 35 pixels/window; distance threshold: 35 pixels). To extract gaze data of the anticipatory phase in the familiarization and test trials, we defined time segments from which gaze data of interest was analyzed. This segment started with the first frame in which the doors flashed and ended 1750 ms later. For these segments three areas of interest (AOIs) were defined. One covered the whole screen (1600 × 900 in Experiment 1 and 1280 × 1024 pixels in Experiment 2). Both, the left- and right-hand doors were each covered by an AOI which was centered in the middle of the door and exceeded its borders by approximately 50 pixels (340 × 310 in Experiment 1 and 360 × 327 pixels each in Experiment 2). We refer to the belief-congruent door as correct door and the other door as incorrect door.

Tobii Studio statistics tool was used to extract the three measures of interest. (1) The location of first fixation was assessed by calculating the time to first fixation on each of three AOIs. The door AOI with the smallest value was classified as firstly fixated. Given that action predictions guiding gaze behavior could have been formed even before the onset of the analysis time window, we decided to include first fixations with a value of 0 ms for one of the door AOIs in our analysis (indicating that the participant was already fixating one of the doors before the chime/flash). A value of 1 was assigned to trials in which the correct door was firstly fixated. For example, if a participant fixated the correct door 500 ms after the onset of the anticipatory phase, and the incorrect door 1200 ms after the onset of the anticipatory phase, this trial was coded as 1. A value of 0 was assigned to trials in which the incorrect door was firstly fixated. Trials, in which no AOI, or just the whole screen but no door was fixated, were coded as 0. (2) To analyze looking time, the total fixation duration on each door during the anticipatory period was calculated. To this end, the sum of total fixation duration (including zeros) was extracted for each door AOI. Trials, in which neither door was fixated during the anticipatory period were coded as missing value. (3) In Experiment 2, as described by Senju et al. (2010), a differential looking score (DLS) was calculated for fixation durations in the total anticipatory period. The DLS was calculated by subtracting the sum of the total duration of fixations on the incorrect door from the sum of the total fixation duration of fixations on the correct door. This was divided by the sum of the total duration of fixations on both doors. The resulting score varies between -1 and 1 (a DLS of 1 indicates a maximum looking bias to the belief-congruent door; a DLS of -1 a maximum looking bias to the other doors; a DLS of 0 can be interpreted as no looking bias to either door). Trials, in which neither the correct nor the incorrect door were fixated, were classified as missing value.

**Analysis of side preferences depending on direction of transfer**

**Experiment 1**. The marginally significant interaction between direction of transfer and door, *F*(1,18) = 3.56, *p* = .076, η*_p_*^2^ = 0.17, resulted from children looking longer to the incorrect door when the object was transferred from the right- to the left-hand box (child’s perspective) than from left to right. We checked whether this could be due to a general bias to look more often at the left- than the right-hand door. For 33 children gaze data was available for both familiarizations and the test trial. The analysis of children’s individual looking pattern regarding these three tasks showed that they tended to look more often at the left door [once (8), twice (15), three times (8)] than at the right door [once (15), twice (8), three times (2)]. The 33 children looked 99 times at one of the doors; 62 times it was to the left and 37 times to the right (*p* = .015, binomial test, chance level of .5). Supplementary Table 1 shows that this was especially pronounced in the test trial of the right-to-left transfer condition in which 82% of children looked at the left door while only 50% did so in the left-to-right transfer condition, χ^2^ (1, *N* = 33) = 3.88, *p*=.049, Φ_Cramer_ = .34. The overall pattern of responses suggests that children had a strong initial left bias which softened somewhat on the second familiarization trial. Furthermore, on the test trial an additional preference for looking at the door above the box, where the object had last been, became operative. This cancels the left bias in the left-to-right transfer direction and enhances it in the right-to left direction. This suggests that these factors need to be carefully controlled if any claims about children looking to where the agent believes the object to be can be reliably drawn. Interestingly, in Southgate et al. (2007) the object was always transferred from left-to-right, therefore, the enhanced left-bias in our right-to-left group could account for part of the discrepancy between our and Southgate et al.’s results.

| Supplementary Table 1  *Experiment 1. Number of children looking at the left or right hand door in both direction of transfer conditions (“+” indicates the correct window)* | | | | | | |
| --- | --- | --- | --- | --- | --- | --- |
| **Transfer at test** | **1^st^ Familiarization** | | **2^nd^ Familiarization** | | **Test Trial** | |
|  | left | right | left | right | left | right |
| left-to-right | 11+ | 5 | 8 | 8+ | 8+ | 8 |
| right-to-left | 10 | 7+ | 11+ | 6 | 14 | 3+ |

**Experiment 2**. For the finally included 17 children of the confirmatory analysis, we checked whether gaze patterns indicated a looking preference towards the left or right side of the screen. None of the three employed measures revealed an effect of direction of transfer (from left to right or reverse). A chi-square test with first fixation score in the test trial as dependent variable indicated no difference between the group who saw the transfer from left to right and the group who saw the transfer from right to left, χ^2^(1, *N* = 17) = 1.43, *p*=.232, Φ_Cramer_ = .29. For looking time, a repeated measure ANOVA with door (correct vs. incorrect) as within-participants factor and direction of transfer (left to right vs. right to left) as between participants factor still showed the significant main effect of door [*F*(1,15) = 6.20, *p* = .025, η*_p_*^2^ = 0.30], but no significant influence of direction of transfer, *F*(1,15) = 1.25, *p* = .281, η*_p_*^2^ = 0.08, and no significant interaction between looking times to the doors and direction of transfer, *F*(1,15) = 0.32, *p* = .579, η*_p_*^2^ = 0.02. Finally, an independent sample *t* test between the two transfer groups with DLS as dependent variable was not significant, *t*(15) = 0.68, *p* = .510, Cohen's *d* = 0.33.

The sample of finally included adults of the confirmatory analysis showed no bias for the left or right side of the screen. A chi-square test with first fixation score in the test trial as dependent variable found only a marginally significant influence of direction of transfer, χ^2^(1, *N* = 54) = 3.71, *p*=.054, Φ_Cramer_ = .26. This marginally significant effect resulted from adults showing more correct anticipations in the test trial when the object was transferred from the right to the left box. This pattern is opposite to what was observed in children in Experiment 1. A repeated measure ANOVA with looking time to door (correct vs. incorrect) as within-participants factor and direction of transfer (left to right vs. right to left) as between participants factor again showed a significant main effect of door, *F*(1,52) = 4.22, *p* = .045, η*_p_*^2^ = 0.08, but neither a significant main effect of direction of transfer, [*F*(1,52) = 1.36, *p* = .248, η*_p_*^2^ = 0.03], nor a significant interaction between door and direction of transfer, [*F*(1,52) = 2.21, *p* = .143, η*_p_*^2^ = 0.04]. Also, an independent samples *t* test between the two transfer groups with DLS as dependent variable was not significant, *t*(52) = -1.65, *p* = .105, Cohen's *d* = -0.45.
